# Initiation of antipsychotics after moving to residential aged care facilities and mortality: a national cohort study

**DOI:** 10.1007/s40520-020-01518-y

**Published:** 2020-03-11

**Authors:** Stephanie L. Harrison, Janet K. Sluggett, Catherine Lang, Craig Whitehead, Maria Crotty, Megan Corlis, Steve Wesselingh, Maria C. Inacio

**Affiliations:** 1 Registry of Senior Australians, Health Ageing Research Consortium, South Australian Health and Medical Research Institute, Adelaide, SA 5000 Australia; 2 Centre for Medicine Use and Safety, Faculty of Pharmacy and Pharmaceutical Sciences, Monash University, Parkville, VIC 3052 Australia; 3 NHMRC Cognitive Decline Partnership Centre, Hornsby Ku-Ring-Gai Hospital, Hornsby, NSW Australia; 4 Department of Rehabilitation, Aged and Extended Care, Flinders University, Rehabilitation Building, Flinders Medical Centre, Bedford Park, SA 5042 Australia; 5 Helping Hand Aged Care, North Adelaide, SA 5006 Australia; 6 South Australian Health and Medical Research Institute, Adelaide, SA 5000 Australia; 7 Liverpool Centre for Cardiovascular Science, University of Liverpool, Liverpool, Merseyside L7 8TX United Kingdom

**Keywords:** Antipsychotics, Dementia, Mortality, Nursing homes

## Abstract

**Background:**

There is a high burden of antipsychotic use in residential aged care facilities (RACFs) and there is concern regarding potential inappropriate prescribing of antipsychotics in response to mild behavioural symptoms. Antipsychotic use has been associated with a higher risk of mortality in community-dwelling older adults with dementia, but few studies have examined associations upon RACF entry.

**Aims:**

To examine associations between incident antipsychotic use and risk of mortality for people with and without diagnosed dementia in RACFs.

**Methods:**

A retrospective cohort study, employing a new-user design (individuals did not receive an antipsychotic 6 months before enrolment) of 265,820 people who accessed RACFs in Australia between 1/4/2008 and 30/6/2015 was conducted. Cox regression models were used to examine adjusted associations between antipsychotic use in the first 100 days of RACF entry and mortality.

**Results:**

In the 100 days after entering care, 29,455 residents (11.1%) were dispensed an antipsychotic. 180,956 (68.1%) residents died [38,249 (14.4%) were related to cerebrovascular causes] over a median 2.1 years (interquartile range 1.0–3.6) follow-up. Of the residents included, 119,665 (45.0%) had a diagnosis of dementia. Incident antipsychotic use was associated with higher risk of mortality in residents with dementia (adjusted hazard ratio 1.20, 95% confidence interval 1.18–1.22) and without dementia (1.28, 1.24–1.31).

**Conclusion:**

Initiation of antipsychotics after moving to RACFs is associated with a higher risk of mortality. Careful consideration of the potential benefits and harms should be given when starting a new prescription for antipsychotics for people moving to RACFs.

**Electronic supplementary material:**

The online version of this article (10.1007/s40520-020-01518-y) contains supplementary material, which is available to authorized users.

## Introduction

Moving to residential aged care facilities (RACFs) can be a distressing time due to the major lifestyle change of moving to an unfamiliar setting, separation from family members, and potential issues regarding privacy and independence [1]. There is a high prevalence of suboptimal medicine prescribing in older adults which have been associated with poorer outcomes including increased hospitalisations and mortality [2]. For older adults living in RACFs, exposure to potentially inappropriate medicines is high and associated with poorer quality of life [3]. Approximately half of older people moving to RACFs in Australia have dementia, [4] and behavioural symptoms such as agitation and aggression, may increase during a move [5]. Pharmacological approaches to managing behaviours for people with dementia include the use of antipsychotics, despite limited effectiveness for this use and the potential for serious adverse events, including mortality [6]. These adverse effects must be considered when determining the appropriateness of prescribing antipsychotics to residents and non-pharmacological approaches are preferred [6, 7].

In Australia, an antipsychotic may be indicated for short-term use among people with dementia when the person has severe agitation and aggression associated with risk of harm, delusions and hallucinations or comorbid pre-existing mental health conditions and does not respond to non-pharmacological approaches [8]. Similar guidelines for prescribing antipsychotics for people with dementia exist in countries such as the United States (US) and Canada [9, 10]. Despite such guidelines, there are concerns that antipsychotics are used inappropriately in response to mild behavioural symptoms such as wandering, insomnia or uncooperativeness [11]. A recent study of Australian RACFs estimated that 22% of residents were prescribed antipsychotics which is similar to the prevalence reported in US long-stay nursing homes [12, 13].

Antipsychotic use for people with dementia has been associated with a higher risk of serious adverse events, including extrapyramidal symptoms, worsening cognitive function, falls, cerebrovascular complications, stroke and premature mortality [14–16] After the publication of a meta-analysis of randomised placebo-controlled trials concluded atypical antipsychotics were associated with a small increased risk of death for people with dementia, the US Food and Drug Administration (FDA) issued an advisory warning in 2005 [17]. This advisory warning was extended to typical antipsychotics in 2008 [18]. Observational studies have suggested the use of typical antipsychotics may have a similar or higher risk of mortality for people with dementia compared to atypical antipsychotics, [19–21] whereas a meta-analysis of randomised placebo-controlled trials did not find any evidence to show an association between typical antipsychotics and mortality risk [22]. When examining associations between antipsychotic use and mortality specifically for people living in RACFs, fewer studies have been specifically undertaken in RACFs, and some variation has been noted. For example, some studies have shown an association between antipsychotic use and higher risk of mortality [23, 24] and others have suggested no association [25, 26]. In this study, we aim to examine associations between incident antipsychotic use and risk of mortality for people with and without diagnosed dementia in RACFs in Australia.

## Methods

### Study design, setting, and data sources

A national study was conducted using the Registry of Senior Australians (ROSA), which captures comprehensive data for all older people who have accessed government-subsidised aged care services in Australia. In ROSA, de-identified data collected during aged care eligibility assessments are linked to information on the types of aged care services the person received, and assessments performed after moving to a RACF [27].

Aged care eligibility assessments are conducted by a team of medical and allied health professionals who collect information in an interview about the person’s sociodemographic characteristics, carer information and physical and psychological health to determine which type of aged care services are appropriate for the individual [28]. The aged care eligibility assessments can record up to ten health conditions, which are coded using a modified International Statistical Classification of Disease and Related Health Problems-Tenth Revision-Australian Modification (ICD-10-AM) classification [28]. In addition, individuals who move to a RACF undergo an assessment to assess their core care needs as a basis for allocating funding [29]. These assessments include detailed information about the person’s physical and psychological health after moving to a RACF including health conditions, activities of daily living (ADLs), behaviour, cognitive impairment and depression. The assessments list up to three health conditions and three mental and behavioural conditions including dementia and use the ICD-10-AM coding system.

The ROSA also contains details of all subsidised medications dispensed for recipients of aged care services via Australia’s national pharmaceutical subsidy schemes (the Pharmaceutical Benefits Scheme (PBS) and Repatriation PBS). Date and primary and other causes of death (classified according to the ICD-10-AM) are available from the linked National Death Index dataset.

### Study participants

The current study includes all people aged 65 years or older or aged 50 years or older if they identified as Aboriginal or Torres Strait Islander, who moved to government-subsidised RACFs between 1/04/2008 (the month after the entry into care assessment was introduced) and 30/06/2015.

Study participants were included if they resided in a RACF for > 100 days and had an assessment within 100 days of entry to aged care. The study adopted a ‘new-user design’ and excluded prevalent users of antipsychotics, which was defined as residents who had an antipsychotic dispensed in the 6 months prior to moving to a RACF. We also excluded residents who were not entitled to access government-subsidised medical and pharmaceutical services to ensure full capture of subsidised medication use for all in the final cohort (*n* = 265,820). See Supplementary Fig. 1 for the study flow diagram.

### Exposure of interest

Antipsychotic use in the first 100 days after RACF entry was the exposure of interest in this study. The World Health Organization (WHO) Anatomical Therapeutic Classification (ATC) System codes were used to identify dispensing for typical antipsychotics (ATC codes N05AA-N05AD, excluding N05AB04, and N05AF) and atypical antipsychotics (N05AE, N05AH, N05AL and N05AX) [30].

### Outcome of interest

The primary outcome of interest was all-cause mortality and the secondary outcome of interest was mortality with cerebrovascular causes (ICD-10-AM codes I60-I69). Residents were followed until death or 31/12/2016, whichever occurred first.

### Covariates

The following covariates were examined: age, sex and co-morbidity score as determined from the Rx-Risk-V, a medication-based co-morbidity measure [31]. The Rx-Risk-V includes 46 co-morbidities and has been validated in older populations and has been to shown to predict 1-year mortality. The following covariates were also examined from the assessment at entry into aged care, [29] which are all ranked on four levels according to independence with the activity or symptoms experienced: ADLs including nutrition, mobility, personal hygiene, toileting, continence, cognitive impairment determined from the Psychogeriatric Assessment Scale-Cognitive Impairment Scale: PAS-CIS, wandering, verbal behavioural symptoms and physical behavioural symptoms and depression determined from the Cornell Scale for Depression (CSD) and diagnosis of depression in previous 12 months.

Dementia was ascertained using the health conditions reported at the aged care eligibility and entry into care assessments (Supplementary Table 1) or if in the 6 months prior to moving to a RACF there was a dispensing history of acetylcholinesterase inhibitor (ATC codes N06DA02, N06DA03 and N06DA04) or memantine (ATC code N06DX01).

### Statistical analyses

Descriptive statistics are presented by whether the residents were dispensed an antipsychotic in the first 100 days after moving to a RACF with frequencies and percentages for categorical variables and means and standard deviations (SDs) or medians and inter-quartile ranges (IQR) for continuous variables. The Kaplan–Meier method was used to estimate median survival time after the first 100 days of living in a RACF. Cox multivariate regression models were used to estimate the risk of all-cause mortality associated with an antipsychotic use in the first 100 days after moving to a RACF compared with no antipsychotic use in the first 100 days for all residents and stratified by dementia status [32]. Age- and sex-adjusted hazard ratios (HRs) and 95% confidence intervals were estimated. Final models were adjusted for all covariates described in the previous section. Proportional hazard assumptions were tested based on Schoenfeld residuals after fitting the Cox model. Analyses were repeated to compare outcomes among residents exposed to typical versus atypical antipsychotics and to examine mortality with cerebrovascular causes as a secondary outcome. Statistical analyses were performed using Stata v.15.0 (Stata Corp LP, College Station, TX, USA).

## Ethical approval

This study received ethical approval from the University of South Australia (Protocol ID: 200489; Evaluating Healthy Ageing in Australia, 1997–2017) and from the Australian Institute of Health and Welfare’s Ethics Committee (EO2018/1/418; Evaluating Healthy Ageing in Australia, 2002–2017). As the data were de-identified, informed consent was not required in line with sought ethical approvals.

## Results

### Characteristics of the study cohort

Of the 265,820 residents included in this study, 65.0% were females, the mean age was 84.4 (SD = 6.9) years, 45.0% had diagnosed dementia and the median number of co-morbidities was 5 (IQR = 3–7). The median follow-up of this cohort was 2.1 years (IQR = 1.0–3.6). In the cohort, 11.1% (*n* = 29,455) were newly dispensed at least one antipsychotic in the first 100 days after moving to a RACF. Of these residents, 15.2% (*n* = 4,469) had only typical antipsychotic(s) dispensed, 76.8% (22,617) had only atypical antipsychotic(s) dispensed, and 8.0% (*n* = 2,369) had both typical and atypical antipsychotics dispensed which could reflect concomitant use or switching. Of the residents who received antipsychotic(s), 76.1% were living with dementia compared to 41.2% of residents who did not receive antipsychotic(s), Table 1. Of the residents living with dementia 18.7% (*n* = 22,408) received an antipsychotic compared to 4.8% (*n* = 7,047) of residents without dementia. The prevalence of use of each antipsychotic is shown in Supplementary Table 2. Haloperidol was the most common typical antipsychotic dispensed (87.8% of residents dispensed typical antipsychotic(s) only) and risperidone was the most common atypical antipsychotic dispensed (77.8% of residents dispensed atypical antipsychotic(s) only).

**Table 1:** Study participant characteristics and outcomes for all residents and stratified by incident antipsychotic use in first 100 days after moving to a residential aged care facility

	All residents	No antipsychotic(s) dispensed	At least one antipsychotic dispensed
Total, *N* (%)	265,820 (100)	236,365 (88.9)	29,455 (11.1)
Age (years), mean (SD)	84.4 (6.9)	84.6 (6.9)	82.8 (7.0)
Female, *n* (%)	172,815 (65.0)	156,506 (66.2)	16,309 (55.4)
Dementia, *n* (%)	119,665 (45.0)	97,257 (41.2)	22,408 (76.1)
Medication-based co-morbidity score, median (IQR)	5 (3–7)	5 (3–7)	4 (2–6)
Medication-based co-morbidity score > 5, *n* (%)	113,164 (42.6)	103,578 (43.8)	9,586 (32.5)
Cardiovascular medications			
IHD (angina or hypertension), *n* (%)	126,631 (47.6)	114,615 (48.5)	12,016 (40.8)
Antihypertensives, *n* (%)	177,772 (44.3)	107,128 (45.3)	10,644 (36.1)
Congestive heart failure, *n* (%)	53,050 (20.0)	49,097 (20.8)	3,953 (13.4)
Hyperlipidaemia, *n* (%)	118,308 (44.5)	106,197 (44.9)	12,111 (41.1)
Anticoagulants, *n* (%)	51,204 (19.3)	47,150 (20.0)	4,054 (13.8)
Antiplatelets, *n* (%)	104,703 (39.4)	94,656 (40.1)	10,047 (34.1)
ADLs, *n* (%)^a^			
Nutrition			
1 = Lowest level (independent)	36,126 (13.6)	34,276 (4.5)	1,850 (6.3)
2	90,449 (34.0)	82,489 (34.9)	7,960 (27.0)
3	113,302 (42.6)	98,970 (41.9)	14,332 (48.7)
4 = Highest level (physical assistance in all items)	25,943 (9.8)	20,630 (8.7)	5,313 (18.0)
Mobility			
1 = Lowest level (independent)	21,442 (8.1)	19,232 (8.1)	2,210 (7.5)
2	34,309 (12.9)	31,250 (13.2)	3,059 (10.4)
3	112,823 (42.4)	100,458 (42.5)	12,365 (42.0)
4 = Highest level (physical assistance in all items)	97,246 (36.6)	85,425 (36.1)	11,821 (40.1)
Personal hygiene			
1 = Lowest level (independent)	5,987 (2.3)	5,752 (2.4)	235 (0.8)
2	38,720 (14.6)	36,153 (15.3)	2,567 (8.7)
3	57,394 (21.6)	53,415 (22.6)	3,979 (13.5)
4 = Highest level (physical assistance in all items)	163,719 (61.6)	141,045 (59.7)	22,674 (77.0)
Toileting			
1 = Lowest level (independent)	44,711 (16.8)	42,280 (17.9)	2,431 (8.3)
2	34,748 (24.4)	59,352 (25.1)	5,396 (18.3)
3	35,335 (13.3)	31,216 (13.2)	4,119 (14.0)
4 = Highest level (physical assistance in all items)	121,026 (45.5)	103,517 (43.8)	17,509 (59.4)
Continence			
1 = Lowest level (No incontinence)	89,535 (33.7)	83,513 (35.3)	6,022 (20.4)
2	17,372 (6.5)	15,841 (6.7)	1,531 (5.2)
3	20,300 (7.6)	18,239 (7.7)	2,061 (7.0)
4 = Highest level (Incontinence > 3–4 times/week)	138,613 (52.2)	118,772 (50.3)	19,841 (67.4)
Behaviour, *n* (%)^b^			
Cognitive Skills^c^			
1 = No/minimal impairment (PAS-CIS 0–3)	54,747 (20.6)	52,645 (22.3)	2,102 (7.1)
2 = Mild impairment (PAS-CIS 4–9)	99,122 (37.3)	92,496 (39.1)	6,626 (22.5)
3 = Moderate impairment (PAS-CIS 10–15)	72,920 (27.4)	62,457 (26.4)	10,463 (35.5)
4 = Severe impairment (PAS-CIS 16–21)	39,031 (14.7)	28,787 (12.2)	10,264 (34.9)
Wandering			
1 = None or < 2 days/week	195,175 (73.4)	181,067 (76.6)	14,108 (47.9)
2 = At least 2 days/week	25,857 (9.7)	22,300 (9.4)	3,557 (12.1)
3 = At least 6 days/week	12,894 (4.9)	10,767 (4.6)	2,127 (7.2)
4 = ≥ Twice/day 6 days/week	31,894 (12.0)	22,231 (9.4)	9,663 (32.8)
Verbal behavioural symptoms			
1 = None or < 2 days/week	78,956 (29.7)	74,920 (31.7)	4,036 (13.7)
2 = At least 2 days/week	52,723 (19.8)	48,123 (20.4)	4,600 (15.6)
3 = At least 6 days/week	44,436 (16.7)	40,274 (17.0)	4,162 (14.1)
4 = ≥ Twice/day 6 days/week	89,705 (33.8)	73,048 (30.9)	16,657 (56.6)
Physical behavioural symptoms			
1 = None or < 2 days/week	124,862 (47.0)	118,743 (50.2)	6,119 (20.8)
2 = At least 2 days/week	44,649 (16.8)	39,997 (16.9)	4,652 (15.8)
3 = At least 6 days/week	28,373 (10.7)	24,777 (10.5)	3,596 (12.2)
4 = ≥ Twice/day 6 days/week	67,936 (25.6)	52,848 (22.4)	15,088 (51.2)
Depression			
1 = No or minimal symptoms (CSD 0–8)	141,610 (53.3)	128,792 (54.5)	12,818 (43.5)
2 = Mild, moderate or major, no diagnosis (CSD 9–13 or CSD 14–38 and no diagnosis)	73,424 (27.6)	64,494 (27.3)	8,930 (30.3)
3 = Moderate and diagnosis (CSD 14–18 and diagnosis)	28,662 (10.8)	24,837 (10.5)	3,825 (13.0)
4 = Major and diagnosis (CSD 19–38 and diagnosis)	22,124 (8.3)	18,242 (7.7)	3,882 (13.2)
Outcomes			
All deaths within the study period, *n* (%)	180,956 (68.1)	159,215 (67.4)	21,741 (73.8)
Deaths within the study period with cerebrovascular causes, *n* (%)	38,249 (14.4)	33,535 (14.2)	4,714 (16.0)
Follow up time (years), Median (IQR)	2.1 (1.0–3.6)	2.1 (1.1–3.7)	1.7 (0.7–3.2)

Cardiovascular medication use defined using the Rx-Risk-V

*ADLs* activities of daily living, *CSD* Cornell Scale for Depression, *IHD* ischaemic heart disease, *PAS-CIS* Psychogeriatric Assessment Scale-Cognitive Impairment Scale

^a^From the entry in to aged care assessment ADLs domain

^b^From the entry in to aged care assessment behaviour domain

^c^Assessment provides additional criteria if PAS-CIS was not deemed appropriate

### Any antipsychotic use and risk of all-cause mortality

Of the residents who received antipsychotic(s) in the first 100 days after moving to a RACF, 73.8% died before the end of the follow-up period compared to 67.4% of residents not dispensed an antipsychotic.

After adjusting for age, sex, co-morbidity score, ADLs, cognitive impairment and behaviour needs, having antipsychotic(s) dispensed in the first 100 days after moving to a RACF was associated with a higher risk of mortality compared with those who had no antipsychotic dispensed (adjusted hazard ratio 1.22, 95% confidence interval 1.20–1.24). This higher risk of mortality was consistent for residents without dementia (1.28, 1.24–1.31) and residents with dementia (1.20, 1.18–1.22), Table 2.

**Table 2:** Risk of mortality associated with incident antipsychotic medication use within 100 days after moving to a residential aged care facility [Hazard ratios (HR) and 95% confidence intervals (CI)]

	All-cause mortality		Mortality with cerebrovascular causes	
	HR (95% CI)^a^	HR (95% CI)^b^	HR (95% CI)^a^	HR (95% CI)^b^
All participants (*n* = 265,820)				
Any antipsychotic	1.31 (1.29, 1.33)	1.22 (1.20, 1.24)	1.33 (1.29, 1.37)	1.17 (1.13, 1.21)
Typical	1.43 (1.39, 1.48)	1.28 (1.24, 1.33)	1.43 (1.33, 1.54)	1.19 (1.11, 1.28)
Atypical	1.25 (1.23, 1.28)	1.17 (1.15, 1.19)	1.29 (1.25, 1.34)	1.15 (1.11, 1.19)
Typical vs. atypical	1.16 (1.12, 1.21)	1.12 (1.08, 1.16)	1.13 (1.05, 1.23)	1.06 (0.98, 1.15)
Dementia (*n* = 119,665)				
Any antipsychotic	1.27 (1.24, 1.29)	1.20 (1.18, 1.22)	1.19 (1.15, 1.24)	1.15 (1.10, 1.20)
Typical	1.23 (1.18, 1.29)	1.14 (1.10, 1.20)	1.25 (1.14, 1.37)	1.16 (1.06, 1.27)
Atypical	1.23 (1.21, 1.25)	1.17 (1.15, 1.19)	1.17 (1.12, 1.22)	1.13 (1.09, 1.18)
Typical vs. atypical	1.04 (0.99, 1.09)	1.02 (0.97, 1.07)	1.10 (0.99, 1.21)	1.06 (0.96, 1.17)
No dementia (*n* = 146,155)				
Any antipsychotic	1.39 (1.35, 1.43)	1.28 (1.24, 1.31)	1.63 (1.54, 1.74)	1.30 (1.22, 1.39)
Typical	1.79 (1.70, 1.89)	1.54 (1.46, 1.63)	1.74 (1.55, 1.96)	1.26 (1.21, 1.42)
Atypical	1.26 (1.22, 1.30)	1.17 (1.13, 1.21)	1.56 (1.46, 1.67)	1.28 (1.19, 1.37)
Typical vs. atypical	1.41 (1.33, 1.51)	1.33 (1.25, 1.42)	1.12 (0.98, 1.28)	1.03 (0.90, 1.18)

^a^Adjusted for age and sex

^b^Adjusted for age, sex, co-morbidity score and activity limitations, verbal and physical behavioural symptoms, wandering, depression and cognitive impairment

### Typical and atypical antipsychotics and risk of all-cause mortality

Typical (1.28, 1.24–1.33) and atypical (1.17, 1.15–1.19) antipsychotic use was independently associated with a higher risk of mortality in the cohort compared to those with no antipsychotics dispensed. Typical antipsychotic use was associated with a higher risk of mortality compared with residents dispensed atypical antipsychotics only (1.12, 1.08–1.16).

For residents with dementia, being dispensed typical antipsychotic(s) (1.14, 1.10–1.20) and atypical antipsychotic(s) (1.17, 1.15–1.19) were independently associated with a comparable higher risk of mortality. For residents without dementia, typical antipsychotic use was associated with a higher risk of mortality compared with residents dispensed atypical antipsychotics only (1.33, 1.25–1.42).

### Antipsychotic use and risk of cerebrovascular mortality

In the cohort, 14.4% died with cerebrovascular causes (16.0% for residents dispensed antipsychotic(s) vs. 14.2% for residents not dispensed an antipsychotic). Antipsychotic use was associated with a higher risk of mortality with cerebrovascular causes (1.17, 1.13–1.21). These associations were similar for atypical and typical antipsychotics and for residents with and without dementia, Table 2.

## Discussion

This national study utilised data collected from all older people moving to RACFs in Australia and demonstrated that incident use of antipsychotics was associated with a higher risk of all-cause mortality for residents with and without dementia. Typical and atypical medications were independently associated with a higher risk of all-cause mortality and mortality with cerebrovascular causes. For residents without dementia, there was a higher risk of mortality associated with the use of typical antipsychotics compared to the use of atypical antipsychotics. Some of the association between antipsychotics and mortality in this study was explained by the characteristics and health conditions of residents receiving antipsychotics, but the final models were adjusted for factors which may influence both antipsychotic use and mortality. In this study, 21,741 residents who newly received antipsychotics after moving to a RACF died within the study period. After adjusting for many potential confounding factors, the study showed a 22% higher risk of mortality associated with incident antipsychotic use. Extrapolation of these findings to the broader Australian population suggests 3921 deaths (95% confidence interval: 3624–4208) among residents of Australian RACFs could potentially be associated with the use of antipsychotics over 2 years.

Our findings are consistent with other observational studies and randomised controlled trials that have shown an association between antipsychotic use and higher risk of mortality for community-dwelling people with dementia [14, 17, 20, 21]. When examining the association between antipsychotics and mortality specifically for people living in RACFs, fewer studies have been conducted with some studies reporting no effect [25, 26, 33, 34]. Variations in study design should be considered as a possible contributing factor to inconsistencies. Some studies which have not found an association between antipsychotic use and mortality in RACFs have been conducted with relatively small sample sizes, which may be underpowered to detect an association and others have not employed a new-user design [25, 26, 33, 34]. A new-user design is preferable because including prevalent users of antipsychotics may introduce a survivor bias [35]. In the current study, we were able to employ a new-user design by excluding people who had been dispensed an antipsychotic in the 6 months before moving to a RACF.

We determined that typical and atypical antipsychotic use were associated with a higher risk of cerebrovascular mortality in our cohort which is also in line with findings of previous studies [36]. Different mechanisms have been proposed to explain the higher risk of mortality associated with antipsychotic use. One such mechanism is that antipsychotics have both direct effects on the vascular system (e.g. changes in blood pressure) and indirect effects (e.g. impaired endothelial function) [37].

In the current study, there was a higher proportion of residents dispensed antipsychotics living with dementia compared to those not dispensed antipsychotics. This is consistent with antipsychotics being utilised in RACFs to manage behavioural symptoms such as aggression and agitation, which may be more commonly experienced by people with dementia, often referred to as behavioural and psychological symptoms of dementia or BPSD [38]. However, there may be various other causes for the behaviours including responses to the change in environment, unmet needs (e.g. pain), delirium, or other external factors rather than being solely attributable to the underlying pathology of dementia [5, 38]. Antipsychotics are only indicated for people with dementia when the person has severe agitation and aggression associated with risk of harm, delusions and hallucinations or comorbid pre-existing mental health conditions [8]. Non-pharmacological approaches tailored to the person’s preferences, skills and abilities should be trialled as a first-line approach [32]. For instance, recent research has suggested functional-analysis based interventions should be trialled as a first-line approach for behavioural symptoms for people with dementia due to similar effects for behavioural symptoms as pharmacological approaches and lack of associated adverse events [7]. Antipsychotics are sometimes prescribed for short-term use in people experiencing distress associated with delirium and when symptoms cannot be resolved with non-pharmacological strategies, however, caution is advised as these medicines can also worsen delirium in older people [39].

Therapeutic use of music and/or dancing, support and counselling, reminiscence therapy or massage may also be beneficial, but approaches should always be person-centred [32].

After moving to a RACF, many residents consult a new general practitioner (GP) and medications may be dispensed by a different pharmacist [40]. Practitioners who have not had contact with the resident before may not be familiar with the usual behavioural symptoms of the resident which may result in potential overuse of antipsychotics. If antipsychotic use is deemed necessary, individuals receiving antipsychotics should be monitored regularly, and an appropriate withdrawal strategy should be considered. Guidelines provide recommendations about assessing risks and benefits of antipsychotics, a plan to taper and withdraw the medication both in instances of no response and when an adequate response is seen [9]. It is possible to withdraw antipsychotics in RACFs without worsening behaviours or an increase in adverse events using multidisciplinary approaches such as re-education and training of care staff and personalised deprescribing protocols [41–43].

### Strengths and limitations of this study

This study utilised a large, nationally representative cohort of all people who accessed government-subsidised RACFs in Australia over a 7-year period. The study included comprehensive information collected by trained professionals about individuals accessing the aged care sector and their pharmaceutical and mortality information (including cause-specific mortality). Due to the universal healthcare system in Australia, the study captures all subsidised antipsychotics dispensed in the first 100 days of RACF entry and all deaths are captured with the National Death Index.

This is one of few studies which has been able to examine incident antipsychotic use after moving to residential aged care. By starting the study at 100 days post-entry to a RACF we have minimised immortal time bias, however, we recognise there may be differences in the total amount of antipsychotic exposure in the 100 days before study entry and we did not assess antipsychotic exposure post-study entry [44]. A major strength of the study over some of the existing claims-based data studies is the inclusion of clinical information including specific resident behavioural symptoms and cognitive assessment results that often are lacking in claims-based data studies. This meant that we could adjust for symptom severity and minimise the risk of confounding.

There are several limitations to the study. One limitation is that medicines which are supplied to hospital inpatients are not PBS-subsidised and only certain Australian hospitals are able to supply PBS-subsidised medicines on discharge. Therefore, the first supply of an antipsychotic may appear in the 100-day period for residents moving to a RACF immediately after a hospital stay where an antipsychotic was commenced. We did not have the indication for antipsychotic use, dose or frequency of use so these could not be examined in this study. In this study, 4.8% of people without a diagnosis of dementia received antipsychotic(s). We could not further explore why people without dementia were prescribed antipsychotics, but this may be due to other severe mental health conditions (e.g. schizophrenia) that could not be captured in this dataset as other mental health conditions (e.g. depression) are grouped together with more severe mental health conditions on aged care assessments. This could also indicate off-label and potentially inappropriate use or undiagnosed dementia. Because we lacked data on indication and in-hospital use, we were also unable to ascertain if the antipsychotic was prescribed for short term treatment (e.g. for symptoms of delirium) or intended for long-term regular use. With using claims-based data we do not have information on medication adherence, but because the majority of residents require assistance with medication management and administration from staff, nonadherence is likely to be minor in this population, although it is unknown if antipsychotics that were dispensed to residents were administered when needed or regularly. Finally, because of the observational nature of our study, while we have adjusted our risk estimate models for many potential confounding factors of the association of antipsychotic use and mortality, there may be residual confounding affecting our estimates.

## Conclusion

In this nationally representative study of people moving to RACFs in Australia, incident antipsychotic use after entering care was associated with a higher risk of mortality. After adjusting for many potential confounding factors, a 20% higher risk of mortality for residents with dementia and a 28% higher risk of mortality for residents without dementia were identified. This adds to the body of evidence to suggest prescription of antipsychotics for people moving to RACFs should only be considered when the person is at risk of harm and/or when other non-pharmacological approaches have failed. The results of this study suggest entry to RACFs could be a critical time for targeting non-pharmacological interventions to support people during their transition to aged care to help avoid the use of antipsychotics.

## Electronic supplementary material

Below is the link to the electronic supplementary material.

Supplementary file1 (DOCX 40 kb)

## References

[1] Bradshaw SA, Playford ED, Riazi A (2012). Living well in care homes: a systematic review of qualitative studies. Age Ageing.

[2] Onder G, Bonassi S, Abbatecola AM (2014). High prevalence of poor quality drug prescribing in older individuals: a nationwide report from the Italian Medicines Agency (AIFA). J Gerontol Ser A.

[3] Harrison SL, O'Donnell LK, Bradley CE, Milte R, Dyer SM, Gnanamanickam ES, Liu E, Hilmer SN, Crotty M (2018). Associations between the drug burden index, potentially inappropriate medications and quality of life in residential aged care. Drugs Aging.

[4] Harrison SL, Lang C, Whitehead C (2019). Trends in prevalence of dementia for people accessing aged care services in Australia. J Gerontol Ser A.

[5] NHS England (2017) Optimising treatment and care for people with behavioural and psychological symptoms of dementia. https://medicine.exeter.ac.uk/media/universityofexeter/medicalschool/pdfs/nhs-dementia-best-practice-guide.pdf. Accessed 1 July 2019

[6] Kirkham J, Sherman C, Velkers C (2017). Antipsychotic use in dementia. Can J Psychiatry.

[7] Dyer SM, Harrison SL, Laver K (2018). An overview of systematic reviews of pharmacological and non-pharmacological interventions for the treatment of behavioral and psychological symptoms of dementia. Int Psychoger.

[8] The Royal Australia AND New Zealand College of Psychiatrists (2016) Antipsychotic medications as a treatment of behavioural and psychological symptoms of dementia. https://www.ranzcp.org/files/resources/college_statements/practice_guidelines/pg10-pdf.aspx. Accessed 5 Mar 2020

[9] Reus VI, Fochtmann LJ, Eyler AE (2016). The American Psychiatric Association practice guideline on the use of antipsychotics to treat agitation or psychosis in patients with dementia. Am J Psychiatry.

[10] Bjerre LM, Farrell B, Hogel M (2018). Deprescribing antipsychotics for behavioural and psychological symptoms of dementia and insomnia: evidence-based clinical practice guideline. Can Fam Phys.

[11] Edge L (2009). Antipsychotic drugs for dementia: a balancing act. Lancet Neurol.

[12] Westbury J, Gee P, Ling T (2019). More action needed: Psychotropic prescribing in Australian residential aged care. Austral N Zeal J Psychiatry.

[13] Department of Health and Human Services (2016) Update Report on the National Partnership to Improve Dementia Care in Nursing Homes. Centers for Medicare and Medicaid Services. https://www.cms.gov/Medicare/Provider-Enrollment-and-Certification/SurveyCertificationGenInfo/Policy-and-Memos-to-States-and-Regions-Items/Survey-and-Cert-Letter-16-28. Accessed 5 Mar 2020

[14] Ballard C, Hanney ML, Theodoulou M (2009). The dementia antipsychotic withdrawal trial (DART-AD): long-term follow-up of a randomised placebo-controlled trial. Lancet Neurol.

[15] Rochon PA, Stukel TA, Sykora K (2005). Atypical antipsychotics and Parkinsonism. JAMA Intern Med.

[16] Vigen CLP, Mack WJ, Keefe RSE (2011). Cognitive effects of atypical antipsychotic medications in patients with Alzheimer's disease: outcomes from CATIE-AD. Am J Psychiatry.

[17] Schneider LS, Dagerman KS, Insel P (2005). Risk of death with atypical antipsychotic drug treatment for dementia: meta-analysis of randomized placebo-controlled trials. JAMA.

[18] Dorsey ER, Rabbani A, Gallagher SA (2010). Impact of FDA black box advisory on antipsychotic medication use. Arch Intern Med.

[19] Wang PS, Schneeweiss S, Avorn J (2005). Risk of death in elderly users of conventional vs. atypical antipsychotic medications. N Engl J Med.

[20] Schwertner E, Secnik J, Garcia-Ptacek S (2019). Antipsychotic treatment associated with increased mortality risk in patients with dementia. A registry-based observational cohort study. J Am Med Dir Assoc.

[21] Gill SS, Bronskill SE, Normand SL (2007). Antipsychotic drug use and mortality in older adults with dementia. Ann Inter Med.

[22] Hulshof TA, Zuidema SU, Ostelo RWJG (2015). The mortality risk of conventional antipsychotics in elderly patients: a systematic review and meta-analysis of randomized placebo-controlled trials. J Am Med Dir Assoc.

[23] Liperoti R, Onder G, Landi F (2009). All-cause mortality associated with atypical and conventional antipsychotics among nursing home residents with dementia: a retrospective cohort study. J Clin Psychiatry.

[24] Huybrechts KF, Rothman KJ, Silliman RA (2011). Risk of death and hospital admission for major medical events after initiation of psychotropic medications in older adults admitted to nursing homes. CMAJ.

[25] Selbaek G, Aarsland D, Ballard C (2016). Antipsychotic drug use is not associated with long-term mortality risk in norwegian nursing home patients. J Am Med Dir Assoc.

[26] Simoni-Wastila L, Ryder PT, Qian J (2009). Association of antipsychotic use with hospital events and mortality among medicare beneficiaries residing in long-term care facilities. Am J Geriatr Psychiatry.

[27] Inacio MC, Bray SCE, Whitehead C (2019). Registry of older south Australians (ROSA): framework and plan. BMJ Open.

[28] Australian Government Australian Institute of Health and Welfare (2002) Aged Care Assessment Program Data Dictionary Version 1.0. https://www.aihw.gov.au/reports/aged-care/aged-care-assessment-program-data-dictionary-versi/contents/table-of-contents. Accessed 01 Sep 2019.

[29] Australian Government Department of Health (2018) Aged Care Funding Instrument (ACFI) User Guide. https://agedcare.health.gov.au/funding/aged-care-subsidies-and-supplements/residential-care-subsidy/basic-subsidy-amount-aged-care-funding-instrument/aged-care-funding-instrument-acfi-user-guide. Accessed 01 June 2019.

[30] World Health Organisation (2013) ATC/DDD Index. https://www.whocc.no/atc_ddd_index/. Accessed 24 Sep 2019.

[31] Sloan KL, Sales AE, Liu CF (2003). Construction and characteristics of the RxRisk-V: a VA-adapted pharmacy-based case-mix instrument. Med Care.

[32] Guideline Adaptation Committee (2016). clinical practice guidelines and principles of care for people with dementia.

[33] Raivio MM, Laurila JV, Strandberg TE (2007). Neither atypical nor conventional antipsychotics increase mortality or hospital admissions among elderly patients with dementia: a two-year prospective study. Am J Geriatr Psychiatry.

[34] Suh GH, Shah A (2005). Effect of antipsychotics on mortality in elderly patients with dementia: a 1-year prospective study in a nursing home. Int Psychogeriatr.

[35] Ray WA (2003). Evaluating medication effects outside of clinical trials: new-user designs. Am J Epidemiol.

[36] Trifiro G, Spina E, Gambassi G (2009). Use of antipsychotics in elderly patients with dementia: do atypical and conventional agents have a similar safety profile?. Pharmacol Res.

[37] Steinberg M, Lyketsos CG (2012). Atypical antipsychotic use in patients with dementia: managing safety concerns. Am J Psychiatry.

[38] Tible OP, Riese F, Savaskan E (2017). Best practice in the management of behavioural and psychological symptoms of dementia. Ther Adv Neurol Disord.

[39] Australian Commision on Safety and Quality in Health Care (2016). Delirium clinical care standard.

[40] Sluggett JK, Ilomaki J, Seaman KL (2017). Medication management policy, practice and research in Australian residential aged care: current and future directions. Pharmacol Res.

[41] Brodaty H, Aerts L, Harrison F (2018). Antipsychotic deprescription for older adults in long-term care: the HALT study. J Am Med Dir Assoc.

[42] Westaway K, Sluggett J, Alderman C (2018). The extent of antipsychotic use in Australian residential aged care facilities and interventions shown to be effective in reducing antipsychotic use: a literature review. Dementia.

[43] Harrison SL, Cations M, Jessop T (2019). Approaches to deprescribing psychotropic medications for changed behaviours in long-term care residents living with dementia. Drugs Aging.

[44] Rothman K, Greenland S, Lash T (2008). Modern epidemiology.

