# Supplementary material for: Initiation of antipsychotics after moving to residential aged care facilities and mortality: a national cohort study
Source: Aging Clin Exp Res. 2020 Mar 11;33(1):95–104. doi: 10.1007/s40520-020-01518-y (PMC7897604; doi:10.1007/s40520-020-01518-y)
Supplement: Supplementary file 1 — Supplementary file1 (DOCX 40 kb) [file 40520_2020_1518_MOESM1_ESM.docx]

**Supplementary Table 1. Aged care assessment codes used to identify residents with dementia.**

| **Aged care assessment codes** | **Condition** |
| --- | --- |
| Dementia | |
| **0500** | **Dementia in Alzheimer’s disease** |
| 0501 | Dementia in Alzheimer’s disease with early onset (<65 yrs) |
| 0502 | Dementia in Alzheimer’s disease with late onset (>65 yrs) |
| 0503 | Dementia in Alzheimer’s disease, atypical or mixed type |
| 0504 | Dementia in Alzheimer’s disease, unspecified |
| **0510** | **Vascular dementia** |
| 0511 | Vascular dementia of acute onset |
| 0512 | Multi-infarct dementia |
| 0513 | Subcortical vascular dementia |
| 0514 | Mixed cortical & subcortical vascular dementia |
| 0515 | Other vascular dementia |
| 0516 | Vascular dementia – unspecified |
| **0520** | **Dementia in other diseases classified elsewhere** |
| 0521 | Dementia in Pick’s disease |
| 0522 | Dementia in Creutzfeldt-Jakob disease |
| 0523 | Dementia in Huntington’s disease |
| 0524 | Dementia in Parkinson’s disease |
| 0525 | Dementia in human immunodeficiency virus (HIV) disease |
| 0526 | Dementia in other specified diseases classified elsewhere |
| **0530** | **Other dementia** |
| 0531 | Alcoholic dementia |
| 0532 | Unspecified dementia (includes presenile & senile dementia) |

Note. In the assessment at entry in to residential aged care dementia is only identified using the codes in bold. All codes are used in the assessment conducted prior to entering residential aged care.

**Supplementary Table 2. Incident antipsychotic medication use within 100 days of entering residential aged care.**

| **Antipsychotic medication (ATC code)** | **Number of residents dispensed the medication,**  **n (%)** |
| --- | --- |
| **Typical only**  **(n=4,469)** | |
| Haloperidol (N05AD01) | 3,923 (87.8) |
| Periciazine (N05AC01) | 292 (6.5) |
| Chlorpromazine (N05AA01) | 215 (4.8) |
| Trifluoperazine (N05AB06) | 27 (0.6) |
| Zuclopenthixol (N05AF05) | 27 (0.6) |
| Fluphenazine (N05AB02) | 11 (0.3) |
| Flupentixol (N05AF01) | 14 (0.3) |
| **Atypical only**  **(n=22,617)** | |
| Risperidone (N05AX08) | 17,599 (77.8) |
| Quetiapine (N05AH04) | 3,046 (13.5) |
| Olanzapine (N05AH03) | 2,937 (13.0) |
| Aripiprazole (N05AX12) | 68 (0.3) |
| Amisulpride (N05AL05) | 34 (0.2) |
| Paliperidone (N05AX13) | 27 (0.1) |
| Ziprasidone (N05AE04) | 8 (0.04) |
| Clozapine (N05AH02) | 10 (0.04) |
| Asenapine (N05AH05) | 1 (<0.01) |

**Supplementary Figure 1. Study flow diagram.**

Excluded people not entitled to government subsidised pharmaceuticals
n=10,942

Excluded people who received antipsychotics in six months prior to entry
n=50,325

Excluded people without an assessment within 100 days of entry
n=3,178

People who lived in residential aged care for >100 days and had an assessment within 100 days of entry, had not received an antipsychotic in the six months prior to entry and were entitled to government subsidised pharmaceuticals
n=265,820

People who lived in residential aged care for >100 days and had an assessment within 100 days of entry and had not received an antipsychotic in the six months prior to entry
n=276,762

People who lived in residential aged care for >100 days 1/4/2008 to 30/6/2015
n=330,265

People who lived in residential aged care for >100 days and had an assessment within 100 days of entry
n=327,087
